# Risk factors for distant metastasis of chondrosarcoma: A population-based study

**DOI:** 10.1097/MD.0000000000035259

**Published:** 2023-09-15

**Authors:** Guang-Hua Deng, Hong Wang, Zhe Tan, Rong Chen

**Affiliations:** a Ya’an Hospital of Traditional Chinese Medicine, Yaan, Sichuan, China.

**Keywords:** chondrosarcoma, metastasis, nomogram, SEER database

## Abstract

Chondrosarcoma is the second largest bone malignancy after osteosarcoma and mainly affects middle-aged adults, where patients with distant metastasis (DM) often have a poor prognosis. Although nomograms have been widely used to predict distant tumor metastases, there is a lack of large-scale data studies for the diagnostic evaluation of DM in chondrosarcoma. Data on patients diagnosed with chondrosarcoma from 2004 to 2015 were obtained from the Surveillance, Epidemiology, and End Results database. Independent risk factors for having DM from chondrosarcoma were screened using univariate and multivariate logistics regression analysis. A nomogram was created to predict the probability of DM from the screened independent risk factors. The nomogram was then validated using receiver operating characteristic curves and calibration curves. A total of 1870 chondrosarcoma patients were included in the study after data screening, of which 157 patients (8.40%) had DM at the time of diagnosis. Univariate and multivariate logistic regression analysis screened four independent risk factors, including grade, tumor number, T stage, and N stage. receiver operating characteristic curves and calibration curves showed good accuracy of the nomogram in both training and validation sets. The current study screened for independent risk factors for DM from chondrosarcoma, which will help clinicians evaluate patients.

## 1. Introduction

Chondrosarcoma is the second largest bone malignancy after osteosarcoma,^[1]^ and related studies show that it accounts for 10% to 15% of bone malignancies,^[2]^ and the incidence of chondrosarcoma cannot be ignored. Chondrosarcoma mainly affects middle-aged people,^[3,4]^ and the most common sites are the proximal femur and the pelvis.^[5–8]^ Currently, the treatment for chondrosarcoma is mainly surgery and chemotherapy,^[9–11]^ which can achieve good results for patients who do not have distant metastasis (DM) in the early stage. However, for patients with chondrosarcoma with DM, the prognosis is poor, and few patients survive surgical treatment and chemotherapy.^[12–14]^ Nomograms are very effective in predicting DM and have been widely used in other fields to predict DM.^[15–18]^ Although studies are currently available that consider high-grade tumors, T3 stage, and large tumor size as independent risk factors for DM in chondrosarcoma.^[19]^ But different data may lead to different conclusions, and using larger data leads to more reliable conclusions, as well as validating the conclusions of previous studies. Therefore, this study selected data from the Surveillance, Epidemiology, and End Results (SEER) database of patients diagnosed with chondrosarcoma from 2004 to 2015 and plotted the nomogram to assess the prediction of DM in chondrosarcoma.

## 2. Method

### 2.1. Patients and statistical collection

Data on patients diagnosed with chondrosarcoma from 2004 to 2015 were obtained from the SEER database. The inclusion criteria were as follows: (1) the patient was a chondrosarcoma patient; (2) complete demographic variables were available, including age, sex, race, and marital status; (3) it had complete clinical and pathological information, including primary tumor location, tumor number, grade, histological type, T stage, N stage, tumor size, and tumor stage. 1870 patients with chondrosarcoma were eventually included in the study through screening.

### 2.2. Statistical analysis

This study was statistically analyzed using SPSS 26.0 and R software (version 4.2.1), and the results were considered statistically significant when *P* < .05. The distribution of variables in the 2 groups was compared using the chi-square test and Fisher exact test.

The 1870 patients included in the study were randomly divided into training and validation sets in a 7:3 ratio. Data from the training set were first subjected to univariate logistic regression analysis in SPSS. Univariate analysis when *P* < .10 variables were included in multivariate logistic regression analysis, and when *P* < .05 variables were identified as independent risk factors. The independent risk factors identified were used to plot the nomogram inside the R software.

The receiver operating characteristic curves were first plotted in the R software and the corresponding area under the curve was calculated to assess the accuracy of the nomogram. In addition, calibration curves were also plotted in the R software to evaluate the accuracy of the nomogram.

## 3. Result

### 3.1. Baseline characteristics of the study population

A total of 1870 patients with chondrosarcoma were screened for inclusion in the study, with 1310 patients divided into the training set and 560 patients divided into the validation set according to the 7:3 ratio. As shown in Table 1, The gender was most commonly male, with 57.56% in the training set and 53.39% in the validation set. The race was most commonly white, with 88.47% in the training set and 85.36% in the validation set. At diagnosis, the most common number of tumors was only one lesion, with 77.18% in the training set and 77.50% in the validation set. Grades were most commonly Grade I and Grade II, accounting for 75.27% of the training set and 76.96% of the validation set. The most common histological types were chondrosarcoma, NOS, which accounted for 76.72% of the training set and 75.00% of the validation set. The most common T stage was T1, which accounted for 58.02% of the training set and 56.43% of the validation set. The most common N stage was N0, which accounted for 98.32% of the training set and 99.11% of the validation set. Meanwhile, the cardinality test or Fisher exact test was performed on the data of both sets and found a completely random distribution in both sets (Table 1).

**Table 1:** Baseline clinical characteristics of chondrosarcoma patients.

Risk factors	Training set	Validation set	*P*
	(n = 1310)	(n = 560)	
Age, years			.411
≤18	30	16	
19–30	137	46	
31–49	379	171	
≥50	764	327	
Sex			.096
Male	754	299	
Female	556	261	
Race			.081
Black	78	49	
White	1159	478	
Other	73	33	
Marital			.247
No	330	127	
Yes	980	433	
Primary site			.379
Axial	593	239	
Limb	652	286	
Other	65	35	
Tumor number			.878
Only one	1011	434	
Other	299	126	
Size, mm			.690
<50	422	183	
50–100	536	218	
>100	352	159	
Grade			.221
I	429	197	
II	557	234	
III	196	66	
IV	128	63	
Histological type			.310
9220	1005	420	
9221	12	10	
9231	140	74	
9240	31	10	
9242	10	3	
9243	112	43	
T stage			.465
T1	760	316	
T2	534	240	
T3 or T4	16	4	
N stage			.192
N0	1288	555	
N1	22	5	

9220, chondrosarcoma, NOS; 9221, juxtacortical chondrosarcoma; 9231, myxoid chondrosarcoma; 9240, mesenchymal chondrosarcoma; 9242, clear cell chondrosarcoma; 9243, dedifferentiated chondrosarcoma.

### 3.2. Incidence and risk factors of DM in chondrosarcoma patients

A total of 157 (8.40%) of the included 1870 patients with chondrosarcoma had DM and 1713 (91.60%) had no DM. As shown in Table 2, the 11 potential factors included were subjected to univariate logistics regression analysis, which revealed age, primary site, tumor number, grade, T stage, N stage, and tumor size as variables associated with DM. The screened correlated variables were included in multivariate logistics regression analysis and found that grade, serial number, T stage, and N stage were independent risk factors for DM of chondrosarcoma (Table 2)

**Table 2:** Univariate and multivariate logistic analyses of distant metastasis in chondrosarcoma patients.

	Univariate analysis	Multivariate analysis		
	*P*	HR	95% CI	*P*
Age	.084	1.150	0.849–1.556	.367
Sex	.588			
Race	.865			
Marital	.192			
Primary site	.649			
Tumor number	.057	2.065	1.152–3.702	.015
Tumor size	<.001	0.927	0.632–1.360	.698
Grade	<.001	1.555	1.197–2.019	.001
Histological type	<.001	1.099	0.96–1.252	.153
T stage	<.001	7.002	3.993–12.280	<.001
N stage	<.001	4.135	1.630–10.488	.003

### 3.3. Nomogram development and validation

**Figure 1.**
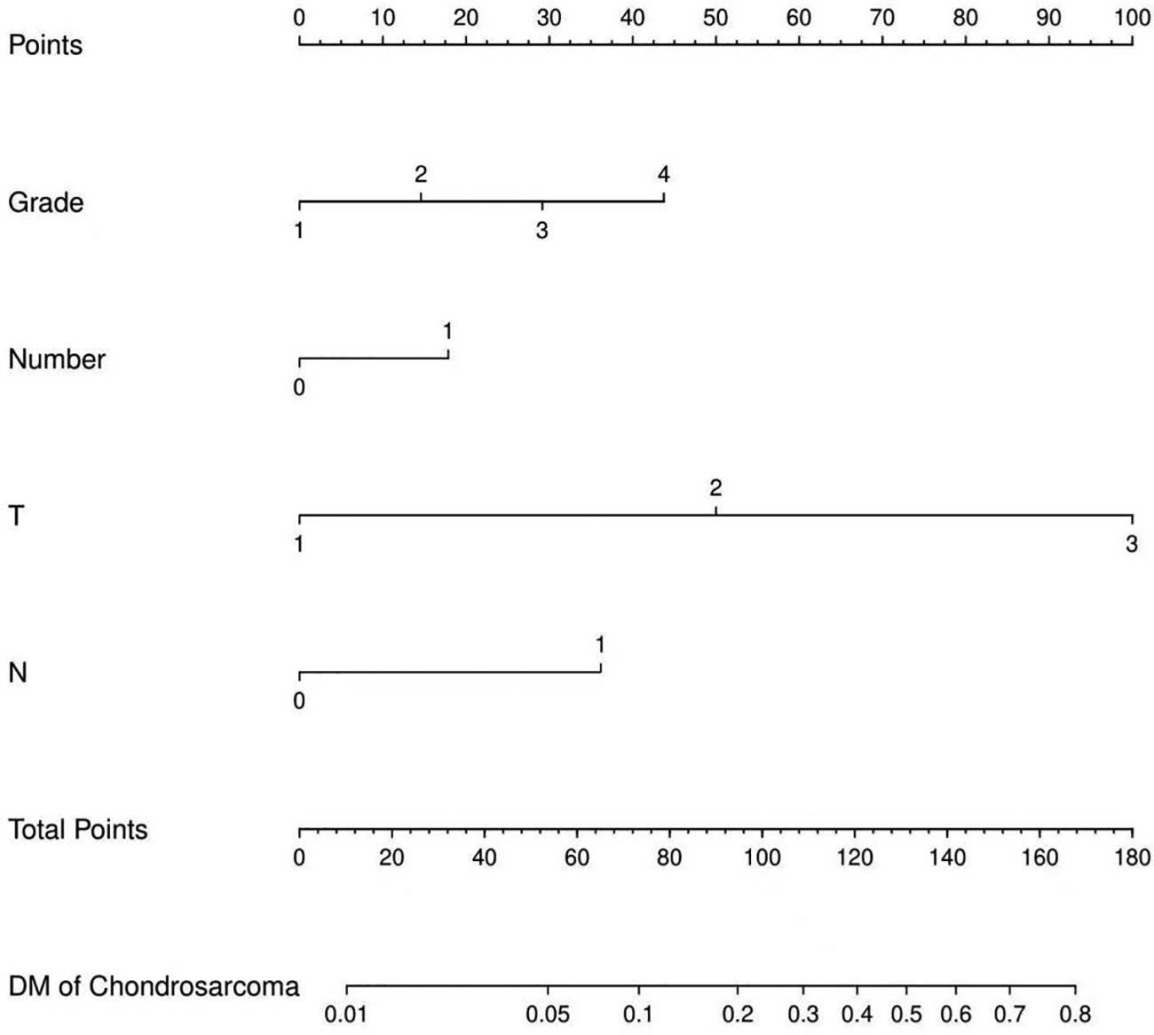
A nomogram to estimate the risk of DM in chondrosarcoma patients. DM = distant metastasis.

**Figure 2.**
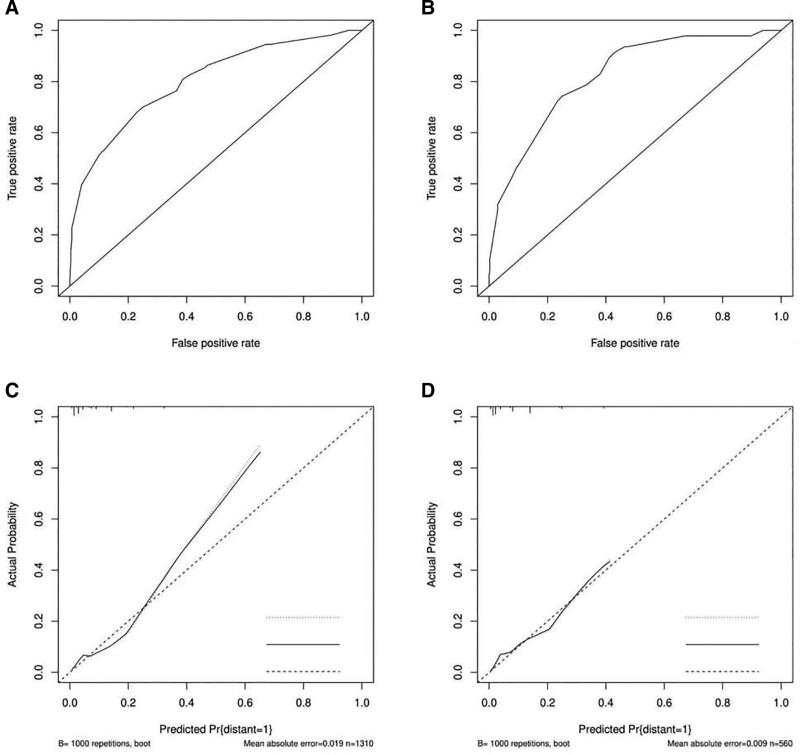
The receiver operating characteristic curve (A), calibration curve (C) of the training set, and the receiver operating characteristic curve (B), calibration curve (D) of the validation set.

The screened independent risk factors were plotted to predict the risk of DM from chondrosarcoma on the nomogram (Fig. 1). Then the receiver operating characteristic curve was plotted for the training and validation sets, and the corresponding curve areas of 0.802 and 0.824 were calculated (Fig. 2A and B). Meanwhile, the calibration curves were plotted to show that the predicted results were in good agreement with the observed results (Fig. 2C and D).

## 4. Discussion

Chondrosarcoma is the second largest bone malignancy after osteosarcoma and has been of interest to bone oncologists. According to related studies, about 8% of chondrosarcoma patients have DM at the time of diagnosis.^[12]^ Giuffrida et al found that patients without DM had a 30-year survival rate more than four times that of patients with DM.^[12]^

There have been many studies on DM from chondrosarcoma. For example, DDX10 and BYSL are presumed to be potential targets for chondrosarcoma,^[20]^ in addition to IDH1/2 and COL2A1 as possible therapeutic targets for chondrosarcoma.^[21]^ However, these studies are based on the molecular level and not on clinical features. In addition, most of these studies are single-center studies and have small sample sizes, resulting in insufficient reliability of these indicators and making it difficult to use them for management with clinicians.

Nomograms have been widely used to predict DM of tumors due to their predictive accuracy.^[15–18]^ Therefore, it is necessary to construct nomograms with clinical features to predict DM of chondrosarcoma.

Currently, a study^[19]^ on clinical characteristics showed that age, primary location, grade, and tumor size were independent risk factors for chondrosarcoma metastasis, while another study^[22]^ suggested that grade, T stage, and tumor size were independent risk factors for DM from chondrosarcoma. The current study included new data and the current data was larger and found the incidence of DM to be 8.40% and identified four important predictors of DM in chondrosarcoma patients: serial number, grade, T stage, and N stage.

Previous studies have confirmed the relationship between chondrosarcoma grade and T stage and DM.^[19]^ However, some studies^[22]^ showed that size was associated with DM, which was related in this study when a univariate analysis was performed, but not when a multifactorial analysis was performed. It was considered that the covariance was due to the existence of covariance between the T stage and size, and the covariance was excluded when the multifactorial analysis was performed. None of the previous studies included the number of tumors at diagnosis in the analysis, but the present study did and found that tumor number was also an independent risk factor. This study showed that the N stage was also an independent risk factor, but other studies^[19,22]^ showed that it was not, which was considered to be due to the different sample sizes, and more and larger samples are needed to verify.

Although the predictive nomograms in this study showed good predictive power, there are some limitations to consider. First, to avoid interference caused by the different diagnostic codes in different years, the included data are only from the clinical data of patients diagnosed with chondrosarcoma in the SEER database from 2004 to 2015, not the data of all chondrosarcoma patients since the establishment of the database. Second, because our study was retrospective, some patient data loss was unavoidable. Third, although the SEER database involves diverse ethnic groups, the United States is still mainly composed of whites and blacks, and there are fewer clinical data records of Asians, which may make the nomogram have certain limitations. Fourth, if the nomogram can be externally detected with large sample data from other databases, the detection result will be more credible, and at the same time, it can also be used to analyze whether the nomogram is universal.

## 5. Conclusion

The current study screened for independent risk factors for DM from chondrosarcoma, which will help clinicians evaluate patients.

## Author contributions

**Conceptualization:** Guang-Hua Deng.

**Data curation:** Guang-Hua Deng, Rong Chen.

**Formal analysis:** Guang-Hua Deng, Rong Chen.

**Funding acquisition:** Guang-Hua Deng.

**Investigation:** Guang-Hua Deng, Zhe Tan, Rong Chen.

**Methodology:** Guang-Hua Deng, Zhe Tan, Rong Chen.

**Project administration:** Guang-Hua Deng.

**Resources:** Guang-Hua Deng, Zhe Tan.

**Software:** Guang-Hua Deng, Zhe Tan.

**Supervision:** Guang-Hua Deng.

**Validation:** Guang-Hua Deng, Hong Wang.

**Visualization:** Guang-Hua Deng, Hong Wang.

**Writing – original draft:** Guang-Hua Deng, Hong Wang.

**Writing – review & editing:** Guang-Hua Deng, Hong Wang.
